# AGE-INCLUSIVE STRATEGIES TO ENHANCE LEARNING AND WORKPLACE ENVIRONMENTS IN U.S. HIGHER EDUCATION

**DOI:** 10.1093/geroni/igad104.0640

**Published:** 2023-12-21

**Authors:** Nina Silverstein, Lauren Bowen, Marla Berg-Weger

## Abstract

This symposium reports on recent research to develop informed strategies to expand age-friendly campus practices related to institutional functions our previous study identified as areas of high-need and broad-impact within an age-friendly campus environment: Teaching and Learning, Personnel, and Student Affairs. The current research aimed to identify barriers and opportunities for age inclusivity as experienced by older campus members (faculty, staff, and students); identify challenges and strategies for implementing age-friendly campus practices; and recommend practical strategies to expand and enhance age-friendly practices. To this end, we drew on data collected from several sources: open-ended responses to the Age-Friendly Inventory and Campus Climate Survey (ICCS) administered to a sample of 23 U.S. members of the Age-Friendly University (AFU) network; responses from 16 focus groups of faculty, administrators/staff, and students; and 18 experts on a nationally selected Delphi panel of experts in higher education. Based on the focus group data, we generated over 50 strategy recommendations within the academic functions that were then evaluated by the Delphi panelists to assess their efficacy, feasibility, likelihood of implementation, and perceived impact. Silverstein will provide an overview of the research objectives, Bowen will discuss what the open-ended responses revealed about perceptions of campus age-inclusivity, Lin and Xu will describe the study samples, Whitbourne will describe the results of the focus group analyses, and Montepare will discuss the final set of recommendations based on the Delphi panel analyses. Berg-Weger will serve as Discussant.

